# *Duganella hordei* sp. nov., *Duganella caerulea* sp. nov., and *Duganella rhizosphaerae* sp. nov., isolated from barley rhizosphere

**DOI:** 10.1007/s10482-025-02160-2

**Published:** 2025-09-01

**Authors:** Katsumoto Kishiro, Nurettin Sahin, Daisuke Saisho, Naoki Yamaji, Jun Yamashita, Yuki Monden, Tomoyuki Nakagawa, Keiichi Mochida, Akio Tani

**Affiliations:** 1 Institute of Plant Science and Resources, Okayama University, Kurashiki, Okayama Japan; 2 Egitim Fakultesi, Mugla Sitki Kocman University, Mugla, Turkey; 3 Graduate School of Environmental, Life, Natural Science and Technology, Okayama University, Okayama, Japan; 4 Faculty of Applied Biological Sciences, Gifu University, Gifu, Japan; 5 RIKEN Center for Sustainable Resource Science, Yokohama, Kanagawa Japan; 6 School of Information and Data Sciences, Nagasaki University, Nagasaki, Japan

**Keywords:** Barley, *Duganella*, Novel species, Rhizosphere

## Abstract

*Duganella* sp. strains R1^T^, R57^T^, and R64^T^, isolated from barley roots in Japan, are Gram-stain-negative, motile, rod-shaped bacteria. *Duganella* species abundantly colonized barley roots. Strains R1^T^, R57^T^, and R64^T^ were capable of growth at 4 °C, suggesting adaptation to colonize winter barley roots. Strains R57^T^ and R64^T^ formed purple colonies, indicating violacein production, while strain R1^T^ did not. Based on 16S rRNA gene sequence similarities, strains R1^T^, R57^T^, and R64^T^ were most closely related to *D. violaceipulchra* HSC-15S17^T^ (99.10%), *D. vulcania* FT81W^T^ (99.45%), and *D. violaceipulchra* HSC-15S17^T^ (99.86%), respectively. Their genome sizes ranged from 7.05 to 7.38 Mbp, and their genomic G+C contents were 64.2–64.7%. The average nucleotide identity and digital DNA–DNA hybridization values between R1^T^ and *D. violaceipulchra* HSC-15S17^T^, R57^T^ and *D. vulcania* FT81W^T^, R64^T^ and *D. violaceipulchra* HSC-15S17^T^ were 86.0% and 33.2%, 95.7% and 67.9%, and 92.7% and 52.6%, respectively. Their fatty acids were predominantly composed of C16:0, C17:0 cyclo, and summed feature 3 (C16:1 ω7c and/or C16:1 ω6c). Based on their distinct genetic and phenotypic characteristics, and supported by chemotaxonomic analyses, we propose that strains R1^T^, R57^T^, and R64^T^ represent novel species within the *Duganella* genus, for which the names *Duganella hordei* (type strain R1^T^ = NBRC 115982^ T^ = DSM 115069^ T^), *Duganella caerulea* (type strain R57^T^ = NBRC 115983^ T^ = DSM 115070^ T^), and *Duganella rhizosphaerae* (type strain R64^T^ = NBRC 115984^ T^ = DSM 115071^ T^) are proposed.

**Supplementary Information:**

The online version contains supplementary material available at 10.1007/s10482-025-02160-2.

## Introduction

The genus *Duganella* is a member of the class *Betaproteobacteria*, order *Burkholderiales*, and family *Oxalobacteraceae*. The first *Duganella* species, *D. zoogloeoides*, was proposed by Hiraishi et al. (1997). Since then, numerous species within the genus have been described. A total of 27 child taxa have been identified; among them, 20 species were validly published under the International Code of Nomenclature of Prokaryotes (ICNP), three were not validly published, and four were identified as incorrect spellings, according to the List of Prokaryotic names with Standing in Nomenclature (LPSN) (https://lpsn.dsmz.de/, Parte et al. 2020). *Duganella* species have been isolated from various environments, including wastewater (Hiraishi et al. 1997), forest soil (Li et al. 2004), rhizosphere soil and the rhizoplane of field-grown sugar cane (Madhaiyan et al. 2013), a subtropical stream (Lu et al. 2020), and flowers (Heo et al. 2022). They are typically Gram-stain-negative, motile, rod-shaped bacteria. Some species produce a purple pigment called violacein (Lu et al. 2022; De León et al. 2023, 2024). Certain species of *Duganella* exhibit plant growth-promoting properties and are capable of solubilizing phosphorus, potassium, and zinc in soils (Verma et al. 2014). Additionally, several *Duganella* strains suppress the plant pathogen *Fusarium graminearum*, likely mediated by their amylolytic, lipolytic, and chitinolytic enzymatic activities (Haack et al. 2016). These reports support the importance of the genera as plant symbiont.

The plant-associated microbiome plays a crucial role in influencing plant health and productivity (Berendsen et al. 2012). Reports on the composition of the microbiome, as well as the isolation and characterization of bacteria from barley are relatively scarce compared to those from rice, wheat, and corn. Yang (2019) reported that seven bacterial isolates belonging to *Bacillus*, *Pseudomonas*, *Paenibacillus*, and *Ensifer* species from the field-grown barley rhizosphere in China demonstrated potential for plant growth promotion and biocontrol of *Fusarium* wilt. Similarly, Timmusk et al. (2011) found that bacterial isolates belonging to *Bacillus* and *Paenibacillus* species from the rhizosphere of wild barley (*H. spontaneum*) growing under stressful conditions at Evolution Canyon in Israel exhibited 1-aminocyclopropane-1-carboxylate deaminase activity, formed biofilms, solubilized phosphorus, and tolerated osmotic stress, highlighting their potential for biotechnological applications. Lewin et al. (2021) investigated the metabolically active bacterial microbiota (SSU rRNA) in two compartments of the rhizosphere of barley and other crops (wheat, rye, and oilseed rape) across different growth stages. They identified the core microbiota specific to each crop species and found that *Massilia* (in barley and wheat) functioned as a keystone taxon. Bulgarelli et al. (2015) examined the structural and functional diversification of the barley root–associated microbiota, revealing that it was primarily composed of bacterial families such as *Comamonadaceae*, *Flavobacteriaceae*, and *Rhizobiaceae.* They also found that the diversity of root-associated bacterial communities was affected by host genotype, and that the barley root–associated microbiota was enriched in functions linked to pathogenesis, secretion, phage interactions, and nutrient mobilization, indicating that microbiota differentiation at the root–soil interface is shaped by the combined action of microbe–microbe and host–microbe interactions.

Double-cropping is an agricultural practice in which two crops are cultivated in different seasons within a single year. In Japan, double-cropping of rice (*Oryza sativa* L.) and barley (*Hordeum vulgare* L.) has long been practiced. Typically, rice is grown during the summer and harvested in autumn, while barley is grown in winter and harvested in spring. The conditions for barley cultivation under this double-cropping system in Japan differ from those in other countries, particularly in terms of soil moisture, as barley generally prefers drier environments. During our investigation of the barley root microbiome under a rice–barley double-cropping system, we found that many colonies from barley root samples formed on Reasoner’s 2A (R2A) agar exhibited similar morphology and purple pigment production, suggesting the dominance of the bacteria of closely related phyla, which were identified as *Duganella* species. Based on phylogenetic analysis, we selected three distinct strains, R1^T^, R57^T^, and R64^T^, for further study. Through phylogenomic analysis, phenotypic assays, and fatty acid profiling, these three strains represent three novel species of the genus *Duganella*, for which the names *Duganella hordei* sp. nov., *Duganella caerulea* sp. nov., and *Duganella rhizosphaerae* sp. nov. are proposed.

## Materials and methods

### Isolation and cultivation

Barley (*H. vulgare* L.) plants were grown in the experimental field of the Institute of Plant Science and Resources, Okayama University, Japan (34° 35′ 29.0″ N/133° 46′ 08.9″ E). The varieties used were ‘Hayakiso 2 (OUJ064)’ and ‘Haruna Nijo (OUJ247)’. These plants were harvested in April 2020, and the soil adhering to the roots was gently shaken off. The roots were washed twice with 35 ml of water in 50 ml tubes. Subsequently, the roots in water were subjected to sonication (30% amplitude, Vibra-Cell™ VC 505, Sonics & Materials, Inc.) for 30 s. The leaves and stems were separately washed with water. Each plant material was then ground individually in 5 ml of water, and their respective diluted macerates were spread on R2A agar. After incubation at 28 °C for 2–3 days, morphologically distinct colonies were selected and transferred onto R2A agar until purified. Strains were routinely cultivated on R2A agar plates and preserved as glycerol stocks at − 80 °C. *D. vulcania* KACC 21471^T^ (= FT81W^T^), obtained from the Korean Agricultural Culture Collection, and *D. violaceipulchra* HSC-15S17^T^ (De León et al. 2023, 2024), provided by the authors, were used for comparative analysis.

### 16S rRNA gene sequencing, identification, and phylogenetic analysis

A single colony of each strain grown on R2A agar was suspended in proteinase K solution (0.2 mg/mL). The suspension was incubated at 60 °C for 20 min, followed by 5 min at 95 °C, and then used as a DNA template for polymerase chain reaction (PCR) reaction. A portion of the 16S rRNA gene was amplified using Quick Taq™ HS DyeMix (Toyobo) and a pair of primers: Eu8f (5′-AGAGTTTGATCCTGGCTCAG-3′) and Eu1492r (5′-GGCTACCTTGTTACGACTT-3′). The 16S rRNA gene sequences were obtained using the BigDye Terminator Cycle Sequencing Kit (Thermo Fisher Scientific) and the primers Eu803r (5′-CATCGTTTACGGCGTGGAC-3′), Eu516f (5′-CCAGCAGCCGCGGTAATAC-3′), and Eu1092f (5′-AAGTCCCGCAACGAGCGCA-3′). Sequence reads were assembled using GENETYX-MAC (Nihon Server Corporation, Tokyo).

To calculate 16S rRNA gene sequence similarity values, global alignment was performed using the algorithm of Myers and Miller (1988) (https://pubmed.ncbi.nlm.nih.gov/3382986/), which is identical to Clustal W alignment with default alignment parameters: gap open penalty = 6.66 and gap extension penalty = 6.66. Alignments were created using Clustal X (v2.1) with the above indicated parameters and we then ran PHYDIT software (Chun 1995) to calculate the similarity matrix from the alignment file. The 16S rRNA gene sequences were compared with those available in the EZBioCloud database (https://www.ezbiocloud.net/, Yoon et al. 2017), and taxonomically identified (Chalita et al. 2024).

To construct the 16S rRNA-based phylogenetic tree, we used 16S rRNA gene sequences extracted from the available genomes using the ContEst16S tool (https://www.ezbiocloud.net/tools/contest16s, Lee et al. 2017). For genomes lacking 16S rRNA gene sequences, we retrieved PCR-based sequences from the NCBI accessions. The phylogenetic tree based on 16S rRNA gene sequences was built with MEGA X software (Kumar et al. 2018). The 16S rRNA gene sequences were aligned using Clustal W (Thompson et al. 1994). Maximum likelihood (ML, Tamura-Nei model) (Felsenstein 1981; Tamura and Nei 1993), neighbor-joining (NJ) (Saitou and Nei 1987), and minimum evolution (ME) (Rzhetsky and Nei 1992) trees were constructed with 1000 replicates as bootstrap values (Felsenstein 1985).

To determine the phylogenomic position, bcgTree (Ankenbrand and Keller 2016) was used to extract the amino acid sequences of 107 single-copy core genes (Dupont et al. 2012) from the genomes of the related 93 strains of *Duganella, Pseudoduganella, Rugamonas*, *Massilia*, and *Janthinobacterium* species. A partitioned maximum likelihood analysis was performed on the concatenated sequences (35,344 positions). The phylogenetic tree was constructed using IQ-TREE version 1.6.12 (Nguyen et al. 2015). The best-fit model (LG+F+I+G4) determined by Model Finder was used for the ML phylogenetic analysis (Kalyaanamoorthy et al. 2017).

### Whole genome sequencing, annotation, and genomic analysis

The genomic DNA of strains R1^T^, R57^T^, and R64^T^, grown in R2A broth at 28 °C for 2 days, was purified following the JGI Bacterial DNA isolation CTAB Protocol (William et al. 2004) and subsequently sequenced on the DNBseq platform, yielding reads of 1.467, 1.236, and 1.392 Gbp, respectively. Genome assembly was performed using Shovill (Seemann 2017) on the Galaxy server (https://usegalaxy.org/, Galaxy Community 2024), and genome annotation was conducted with the DDBJ Fast Annotation and Submission Tool (https://dfast.ddbj.nig.ac.jp/, Tanizawa et al. 2018). Genome completeness and contamination were checked using CheckM (v1.2.3, Parks et al. 2015).

Digital DNA–DNA hybridization (dDDH) analysis was performed in silico using the Type (Strain) Genome Server (TYGS) service (https://tygs.dsmz.de/). The *d*_*4*_ formula was used because it is independent of genome length and therefore robust for use with incomplete draft genomes, as reported by Meier-Kolthoff and Göker (2019). Average nucleotide identity by BLAST (ANIb) was calculated using the JSpeciesWS Online Service (https://jspecies.ribohost.com/jspeciesws, Richter et al. 2016), and pairwise ANIb values were averaged to obtain a single representative value for each genome pair. Average amino acid identities (AAI) were calculated with EzAAI tool v1.2.3 (Kim et al. 2021) with default settings, which uses MMSeqs2 (Kallenborn et al. 2024) for protein comparisons. The pathways/modules encoded in the genomes were analyzed using GenoMaple within the Microbial Genome Database (https://mbgd.nibb.ac.jp/, Uchiyama et al. 2025).

### Phenotypic and chemotaxonomic characterizations

The cells of strains R1^T^, R57^T^, and R64^T^ were stained with nigrosin and observed under a microscope for cell size measurement using ImageJ software (Schneider et al. 2012). Strains R1^T^, R57^T^, and R64^T^ were cultured in R2A broth with varying concentrations of NaCl (0, 0.5, 1, 1.5, 2, 2.5, 3, 3.5, and 4% [w/v]). Additionally, these strains were grown in R2A broth at different temperatures (4, 15, 28, and 37 °C) and across a range of pH values (pH 4.0, 5.0, 6.0, 7.0, 8.0, 9.0, and 10.0), adjusted with buffers described by Lv et al. (2017). To examine the antibiotic and metal resistance, strains R1^T^, R57^T^, R64^T^, *D. vulcania* KACC 21471^T^, and *D. violaceipulchra* HSC-15S17^T^ were cultured on R2A agar supplemented with kanamycin, rifampicin, ampicillin, tetracycline, or chloramphenicol at concentrations of 0.1, 0.5, 1, 5, 25, 50, and 100 µg/mL, as well as NiCl_2_, CoCl_2_, or CuCl_2_ at concentrations of 0.01, 0.05, 0.1, 0.25, 0.5 and 1 mM at 28 °C for 2 days.

Phenotypic assays, including enzymatic activities and carbon source utilization, were conducted using API 20NE strips (bioMérieux, France), and further physiological and carbon source oxidations were determined using GEN III MicroPlates (Biolog Inc., USA), in accordance with the manufacturer’s instructions. Both assays were conducted on strains R1^T^, R57^T^, R64^T^, *D. vulcania* KACC 21471^T^, and *D. violaceipulchra* HSC-15S17^T^. The MicroPlates were incubated at 28 °C for 5 days, and the absorbance at OD590 was measured. To normalize for baseline absorbance and non-metabolic turbidity, the average well development was calculated from the carbon source wells (columns 1–9) to represent overall metabolic activity. Based on this average threshold, each well was classified as positive, weak, or negative. Visual inspection of redox dye color change for scoring was also employed to validate substrate oxidation patterns.

Fatty acid profiles were analyzed at TechnoSuruga Laboratory Co., Ltd. (Shizuoka, Japan). Strains R1^T^, R57^T^, and R64^T^, *D*. *vulcania* KACC 21471^T^, and *D*. *violaceipulchra* HSC-15S17^T^ were cultured in R2A broth at 28 °C. The cells were harvested at the stationary phase by centrifugation at 17,800 × *g* for 5 min at room temperature and washed twice with 0.85% (w/v) NaCl. The prepared samples were frozen at − 80 °C and shipped under refrigerated conditions to TechnoSuruga Laboratory (Shizuoka, Japan) for fatty acid composition analysis. TechnoSuruga Laboratory followed the cellular fatty acid profiling manual in Sherlock Microbial Identification System (version 6.2, MIDI, USA), with calculation method TSBA6 and TSBA6 library.

Strains R1^T^, R57^T^, R64^T^, *D*. *vulcania* KACC 21471^T^, and *D*. *violaceipulchra* HSC-15S17^T^ were cultivated on R2A agar at 28 °C for 2 days, followed by incubation at 4 °C until visible purple pigmentation developed (3–4 days). Approximately 10 mg (wet weight) of bacterial colonies was harvested from the agar surface and suspended in 250 µL of 1-butanol. The suspension was subjected to sonication (30% amplitude, Vibra-Cell™ VC 505, Sonics & Materials, Inc.) for 1 min. Cellular debris was removed by centrifugation at 9100 × *g* for 3 min at room temperature, and the supernatant containing extracted violacein was collected. Commercial violacein (Cayman Chemical, cat. no. 27959) was dissolved in 1-butanol to a final concentration of 0.1 mg/mL and used as a standard in spectrophotometric analyses. Absorbance spectra were recorded from 350 to 900 nm using a microplate reader (DS Pharma Biomedical).

## Results and discussion

### Diversity of the bacterial isolates from barley samples

A total of 103 strains, belonging to 25 genera and 41 species, were isolated from barley (Table S1). Of these, 20 strains were isolated from the leaves, 25 strains from the stems, and 58 strains from the roots. The most frequent isolates were *Staphylococcus* species (8/20 and 15/25 from the leaves and stems, respectively), and *Duganella* species (13/58) from the roots. Most *Duganella* strains exhibited purple coloration. To further investigate this unique characteristic of *Duganella*, three strains, R1^T^, R57^T^, and R64^T^, were selected for study. These strains were chosen due to their phylogenetic divergence, with R57^T^ and R64^T^ exhibiting a purple pigmentation, in contrast to the non-pigmented R1^T^.

### 16S rRNA gene sequence analysis and phylogeny

The 16S rRNA gene sequence similarities between the three strains and their relatives are summarized in Table S2. The 16S rRNA gene sequence analysis indicated that strains R1^T^, R57^T^, and R64^T^ showed the highest similarity to *D. violaceipulchra* HSC-15S17^T^ (99.10%), *D. vulcania* FT81W^T^ (99.45%), and *D. violaceipulchra* HSC-15S17^T^ (99.86%). These values exceeded the 98.7% threshold proposed by Stackebrandt and Ebers (2006) for species delineation. However, Rossi-Tamisier et al. (2015) showed that many of the current bacterial species with validly published names do not conform to this threshold.

The ML, NJ, and ME phylogenetic trees based on 16S rRNA gene sequences (Fig. S1–S3) showed poor resolution with low bootstrap support. Thus, 16S rRNA sequences are inadequate for delineating species within the genera *Duganella*, *Rugamonas*, *Pseudoduganella*, *Massilia*, and *Janthinobacterium* in *Oxalobacteraceae*, as previously reported by Ma et al. (2023). Therefore, phylogenetic analysis should be based on their genome data.

### Genome analysis, phylogeny, and identification

The genome features of the isolates, along with those of the most related species, are summarized in Table S3. Their genomes were characterized by relatively large size (7.05–7.37 Mbp) and high G+C contents (64.2–64.7%).

The ANIb, AAI, and dDDH values among these strains are summarized in Table 1, while those among strains R1^T^, R57^T^, R64^T^, and their related strains are summarized in Table S4. Strains R1^T^ and R64^T^ showed the highest ANIb values with R64^T^ and *D. violaceipulchra* HSC-15S17^T^ (86.4% and 92.7%, respectively). Strain R57^T^ showed a 95.7% ANIb value with *D. vulcania* FT81W^T^. Strains R1^T^ and R64^T^ showed the highest AAI values with R64^T^ and *D. violaceipulchra* HSC-15S17^T^ (87.5% and 87.1%, respectively). Strain R57^T^ showed a 96.9% AAI value with *D. vulcania* FT81W^T^. The dDDH values among all these strains were below 70%, with the highest value observed between *D. vulcania* FT81W^T^ and strain R57^T^ at 67.9%, which remains below the established threshold of 70% for species delineation.

**Table 1:** ANIb, AAI, and dDDH values among strains R1^T^, R57^T^, R64^T^, HSC-15S17^T^, and FT81W^T^

	Strains	Accession No	1	2	3	4	5
*ANIb*							
1	*Duganella violaceipulchra* HSC-15S17^T^	GCF_019166075.1	–				
2	*Duganella* sp. R64^T^	GCA_040364205.1	92.7	–			
3	*Duganella vulcania* FT81W^T^	GCF_009857655.1	89.3	89.4	–		
4	*Duganella* sp. R57^T^	GCA_040362985.1	89.3	89.4	95.7	–	
5	*Duganella* sp. R1^T^	GCF_040369125.1	86.0	86.4	85.8	85.9	–
	Pairwise ANIb values were averaged to obtain a single representative value for each genome pair						
*AAI*							
1	*Duganella violaceipulchra* HSC-15S17^T^	GCF_019166075.1	–				
2	*Duganella* sp. R64^T^	GCA_040364205.1	94.2	–			
3	*Duganella vulcania* FT81W^T^	GCF_009857655.1	90.2	90.4	–		
4	*Duganella* sp. R57^T^	GCA_040362985.1	90.3	90.5	96.9	–	
5	*Duganella* sp. R1^T^	GCF_040369125.1	87.1	87.5	86.6	86.6	–
*dDDH*							
1	*Duganella violaceipulchra* HSC-15S17^T^	GCF_019166075.1	–				
2	*Duganella* sp. R64^T^	GCA_040364205.1	52.6	–			
3	*Duganella vulcania* FT81W^T^	GCF_009857655.1	40.6	40.6	–		
4	*Duganella* sp. R57^T^	GCA_040362985.1	40.1	40.3	67.9	–	
5	*Duganella* sp. R1^T^	GCF_040369125.1	33.2	33.5	32.8	32.4	–

As discussed by Palmer et al. (2020), the conventional cutoff for species delineation typically falls between 95 and 96% for ANI. However, this threshold should be adjusted based on the characteristics of the specific genus, the analytical methods used, and the evolutionary context. Figure S4 presents the correlations between dDDH and ANIb, and between dDDH and AAI, among *Duganella* and *Rugamonas* species. For the correlation between dDDH and ANIb, a cubic regression model yielded a high R-squared value of 0.9964, slightly outperforming the quadratic model (R^2^ = 0.9922, data not shown). Based on the cubic equation, a dDDH value of 70% corresponds to an ANIb of 96.24%, which is notably higher than the ANIb value observed between strain R57^T^ and *D. vulcania* FT81W^T^ (95.7%). A similar trend was observed in the correlation between dDDH and AAI. However, the cubic regression model showed a lower R-squared value of 0.9463 (0.9378 for the quadratic model, data not shown), likely due to greater variability in AAI values. According to the cubic model, a dDDH value of 70% corresponds to an AAI of 98.53%, which is again higher than the AAI between strain R57^T^ and *D. vulcania* FT81W^T^ (96.9%). Taken together, these analyses indicate that strain R57^T^ should be considered a distinct species, based on genus-specific patterns of genomic divergence.

**Fig. 1:**
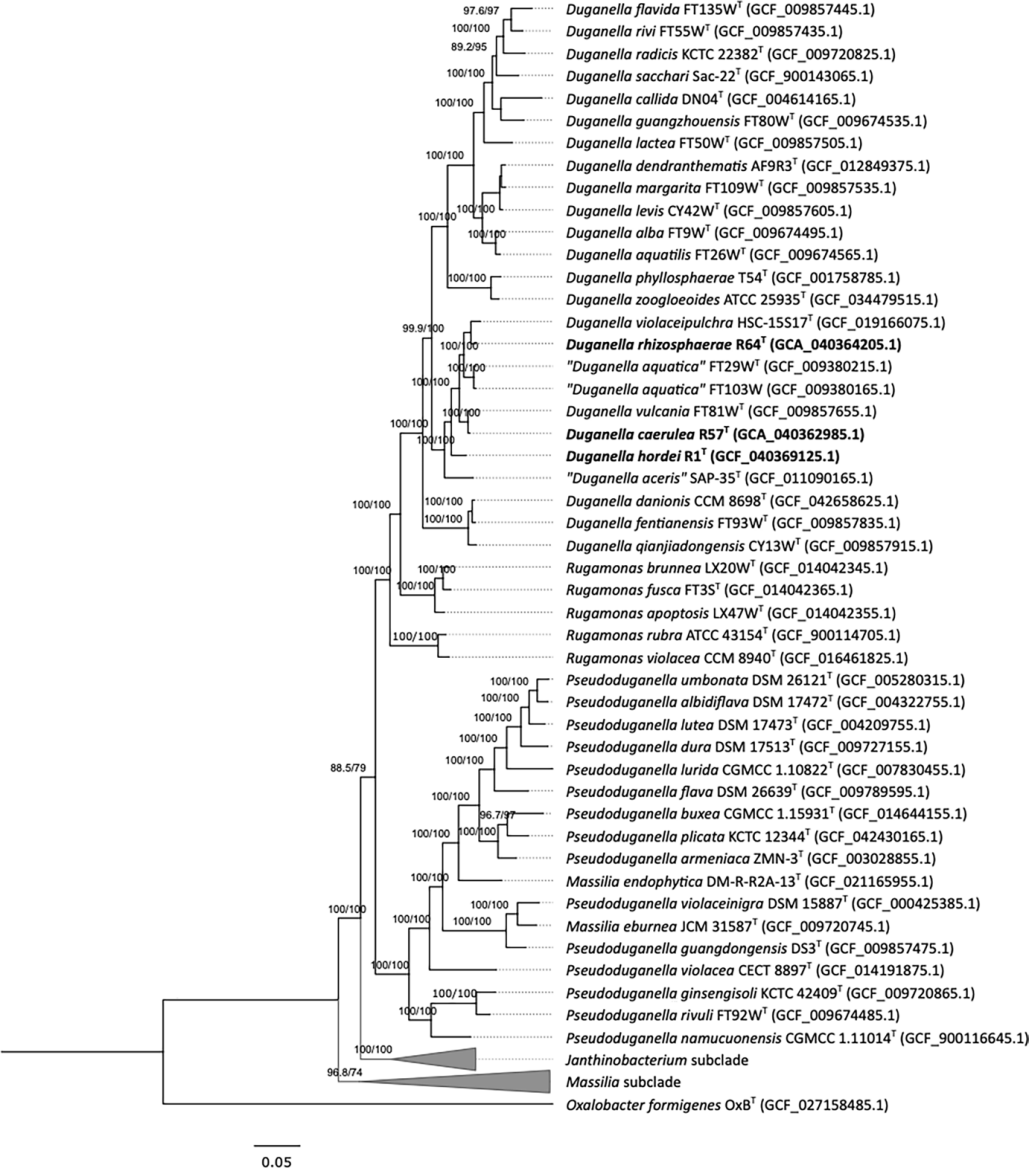
Whole-genome phylogeny based on 107 single-copy core genes. Maximum likelihood analysis was inferred using the LG+F+I+G4 model and rooted by midpoint-rooting. A total of 35,344 amino acid positions were used. Assembly accession numbers are given in parentheses. *Oxalobacter formigenes* OxB^T^ (GCF_027158485) was used as an outgroup. Support values shown on the branches represent SH-aLRT support (%)/bootstrap support (%). Bar, 0.05 substitutions per position

The phylogenomic tree based on the alignment of core 107 single-copy genes among the related 93 strains of *Duganella, Pseudoduganella, Rugamonas*, *Massilia*, and *Janthinobacterium* species including R1^T^, R57^T^, and R64^T^ is presented in Fig. 1, and the list of the genomes are presented in Table S6. Strains R1^T^, R57^T^, and R64^T^ formed a robust monophyletic subclade within the genus *Duganella*, clustering with “*Duganella aquatica*” FT29W^T^, *D. vulcania* FT81W^T^, and *D. violaceipulchra* HSC-15S17^T^ with high bootstrap support. Given their distinct placement and separation from the other *Duganella* species, these strains represent novel species within the genus *Duganella*.

Conversely, our phylogenomic analysis revealed that *M. endophytica* and *M. eburnea* are robustly nested within the *Pseudoduganella* clade with high bootstrap support. This topology contradicts their current taxonomic classification, suggesting that taxonomic verification and possible reclassification as members of the genus *Pseudoduganella* are warranted.

The pathways/modules encoded in the genomes of the isolates were analyzed with GenoMaple (Table S7), and the differential modules are presented in Table S8. GenoMaple analysis revealed notable differences among strains R1^T^, R64^T^, and *D. violaceipulchra* HSC-15S17^T^. The genomes of strains R1^T^ and R64^T^ lacked the cobalt/nickel transport system (M00245) and nickel transport system (M00246), which were present in *D. violaceipulchra* HSC-15S17^T^. Additionally, the copper processing system (M00762) was absent in strains R1^T^ and R64^T^ but present in *D. violaceipulchra* HSC-15S17^T^. Differences were also observed in the multidrug resistance efflux pump AdeABC (M00649) module, which was fully complete (100%) in R64^T^ but absent (0%) in both R1^T^ and *D. violaceipulchra* HSC-15S17^T^, suggesting a potential difference in antibiotic resistance capabilities among the strains. The genome of strain R57^T^ did not encode the sulfonate transport system (M00436) that is present in *D. vulcania* FT81W^T^. Aside from this, many modules that were 100% complete in strain R57^T^ were incomplete in strain *D. vulcania* FT81W^T^.

### Phenotypic and chemotaxonomic characterization

**Fig. 2:**
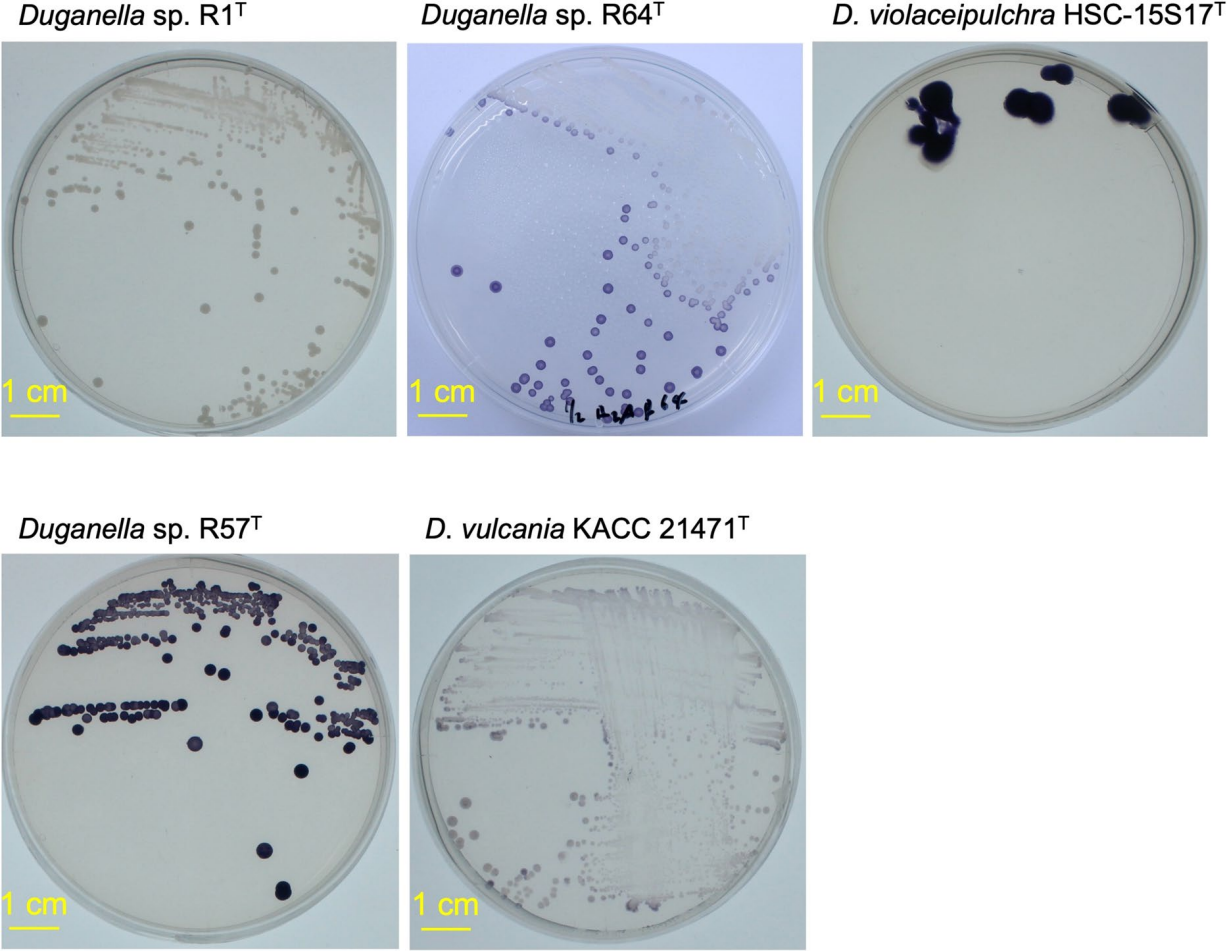
Colony morphology of strains R1^T^, R57^T^, R64^T^, HSC-15S17^T^, and KACC 21471^ T^. Colonies were grown on R2A agar at 28 °C for 2 days, followed by incubation at 4 °C for 3 to 4 days

The cells of strains R1^T^, R57^T^, and R64^T^ measured approximately 0.5 × 1.3 µm, 0.8 × 1.7 µm, and 0.7 × 1.3 µm in size, respectively (Fig. S5). On R2A agar, strain R1^T^ formed white colonies, strain R64^T^ tended to form white colonies at high cell density, whereas it produced purple colonies at lower density, and *D*. *violaceipulchra* HSC-15S17^T^ formed purple colonies. Strain R57^T^ and *D*. *vulcania* KACC 21471^T^ formed purple colonies (Fig. 2). Thus, the violacein production in R64^T^ is regulated by quorum sensing. Colony morphology of strain R1^T^ differed markedly from that of *D*. *violaceipulchra* HSC-15S17^T^, as did the morphology of R64^T^. Additionally, the colony appearance of R57^T^ was distinct from that of *D*. *vulcania* KACC 21471^T^, with R57^T^ exhibiting a deeper purple pigmentation.

The violacein synthesis gene cluster (*vioABCDE*) was found in the genomes of strains R57^T^ and R64^T^ but not in that of strain R1^T^ (Table S7). The 1-butanol extracts of R2A-grown strains R57^T^, R64^T^, *D. vulcania* KACC 21471^ T^, and *D. violaceipulchra* HSC-15S17^T^ cells exhibited absorption peaks at approximately 583 nm. This peak position corresponded to that of standard violacein (Fig. S6). The extract of strain R1^T^ did not show any absorbance in this region.

All three strains grew on R2A broth containing 0 and 0.5% (w/v) NaCl. They also grew on R2A broth at 4, 15, and 28 °C and pH 6.0 to 8.0. The ability to grow at 4 °C indicates that these strains are psychrotrophic. Cold-adaptation genes were identified in the genomes of all three strains: two, two, and three genes encoding cold shock proteins were found in strains R1^T^, R57^T^, and R64^T^, respectively, while one antifreeze protein gene was present in all strains. Additionally, a fatty acid desaturase gene was detected exclusively in strain R57^T^.

To determine whether the observed differences in metal transport and multidrug resistance systems corresponded to phenotypic variation, we conducted antibiotic (Table S9) and heavy metal (Table S10) resistance assays on the isolates. However, little variation in antibiotic and heavy metal resistance was observed among the isolates, and the differences did not manifest at the phenotypic level. Strains R1^T^, R57^T^, and R64^T^ were isolated from the rhizosphere, whereas *D. violaceipulchra* HSC-15S17^T^ was obtained from water dripping from a fern, and *D. vulcania* FT81W^T^ from a subtropical stream. These differences in their habitats may have contributed to the observed genomic variation.

The differential biochemical characteristics analyzed with API 20NE strips and GEN III MicroPlates are listed in Table 2 (all data are summarized in Table S11). Strains R1^T^ and R64^T^ were able to utilize more carbon sources, especially sugars and sugar acids, than *D. violaceipulchra* HSC-15S17^T^, which might explain their adaptation to the rhizosphere environment. Strain R57^T^ can be distinguished from *D. vulcania* KACC 21471^T^ by its inability to oxidize certain amino acids (L-alanine, L-aspartic acid, L-glutamic acid), sugar acids (D-gluconic acid, D-saccharic acid), organic acids (methyl pyruvate, citric acid, α-ketoglutaric acid, β-hydroxy-D,L-butyric acid), and the dipeptide glycyl-L-proline. These results clearly demonstrate the differences in chemotaxonomic phenotypes among the isolates and *D. violaceipulchra* HSC-15S17^T^ and *D. vulcania* KACC 21471^T^.

**Table 2:** Differential biochemical characteristics among strains R1^T^, R64^T^, HSC-15S17^T^, R57^T^, and KACC 21471^T^

Test performed	R1^T^	R64^T^	HSC-15S17^T^	R57^T^	KACC 21471^T^
API 20NE strips					
Reaction/enzyme					
Reduction of nitrates	+	−	−	+	+
Gelatin hydrolysis (protease)	−	−	−	−	+
β-galactosidase	+	−	+	−	+
Assimilation					
Glucose	+	−	−	−	−
L‐arabinose	+	−	−	+	+
D-mannose	+	+	−	+	+
N-Acetyl-D-glucosamine	+	+	−	−	−
Maltose	+	+	−	+	+
Potassium gluconate	+	−	−	+	+
Biolog GENIII					
Dextrin	−	−	−	−	w
D-Maltose	−	−	+	−	w
D-Trehalose	−	−	−	−	w
D-Cellobiose	−	−	+	−	−
Gentiobiose	−	−	+	−	−
D-Turanose	−	−	+	−	−
α-D-Lactose	−	−	−	+	+
D-Melibiose	−	−	−	−	w
N-acetyl-D-galactosamine	−	−	−	+	+
α-D-Glucose	−	−	+	−	w
D-Mannose	+	+	−	+	+
D-Fructose	−	w	−	+	w
3-Methyl glucose	−	−	−	+	−
D-Fucose	−	−	W	−	w
L-Fucose	−	−	−	+	+
L-Rhamnose	−	−	w	w	−
Inosine	−	−	−	+	−
D-sorbitol	−	−	W	−	−
Glycerol	−	−	−	w	−
D-glucose-6-PO4	−	−	+	w	w
D-fructose-6-PO4	−	−	+	−	w
Gelatin	−	−	−	−	w
Glycyl-L-proline	−	−	−	−	+
L-alanine	−	−	−	−	+
L-aspartic acid	−	−	−	−	+
L-glutamic acid	−	−	−	−	+
L-pyroglutamic acid	−	−	−	−	w
L-serine	−	−	−	+	+
Pectin	−	−	−	+	+
D-galacturonic acid	+	+	−	+	+
L-galactonic acid lactone	+	+	−	+	+
D-gluconic acid	−	−	−	−	+
D-glucuronic acid	−	+	+	+	+
Glucuronamide	+	+	−	w	+
D-saccharic acid	−	−	−	−	+
Methyl pyruvate	−	−	−	−	+
D-lactic acid methyl ester	−	−	−	+	+
Citric acid	−	−	−	−	+
α-keto-glutaric acid	−	−	−	−	+
L-malic acid	−	−	+	−	−
Bromo-succinic acid	−	−	+	−	w
Tween 40	−	−	−	−	w
β-Hydroxy-D,L-butyric Acid	−	−	+	−	+
Acetoacetic acid	−	−	w	−	−

Stains: R1^T^. *Duganella* sp. R1^T^; R64T. *Duganella* sp. R64^T^; HSC-15S17^T^. *Duganella violaceipulchra* HSC-15S17^T^; R57^T^. *Duganella* sp. R57^T^; KACC 21471^T^. *Duganella vulcania* KACC 21471^T^. All data were obtained from this study

+, positive; −, negative; w, weak/delayed reaction

Comparison of the fatty acid profiles is summarized in Table 3. Strain R1^T^ exhibited a 7.7% lower proportion of C16:0 and a 5.7% higher proportion of C17:0 cyclo than *D*. *violaceipulchra* HSC-15S17^T^. Strain R57^T^ exhibited a 7.4% lower proportion of C16:0 and a 5% higher proportion of summed feature 3 than *D*. *vulcania* KACC 21471^T^. Strain R64^T^ exhibited a 10% lower proportion of C16:0 and a 13% higher proportion of summed feature 3 compared to *D*. *violaceipulchra* HSC-15S17^T^. The fatty acid profiles of R1^T^ and R64^T^ differed in C17:0 cyclo and summed feature 3, indicating they are also distinct species from each other.

**Table 3:** Fatty acid profiles of strains R1^T^, R64^T^, HSC-15S17^T^, R57^T^, and KACC 21471^ T^

Fatty acid	R1^T^	R64^T^	HSC-15S17^T^	R57^T^	KACC 21471^ T^
C10:0	tr	tr	tr	tr	tr
C10:0 3OH	4.7	4.0	3.2	5.3	4.9
C12:0	6.8	6.7	6.0	7.4	6.9
C13:0 iso	tr				
C14:1 ω5c	tr				
C16:0 iso		tr			
C14:0	tr	1.1	tr	tr	tr
C16:1 ω5c	Tr				
C16:0	37.9	35.6	45.6	35.1	42.5
C17:1 ω7c	Tr			tr	
C17:0 cyclo	18.3	7.4	12.6	16.5	16.5
C17:0	tr				
C18:0	tr	tr	tr	tr	
C19:0 iso		tr			
C20:0	tr	tr			
Summed Feature 3*	27.0	41.1	28.4	30.3	25.3
Summed Feature 8*	3.1	2.6	2.1	3.4	2.7
Summed Feature 9*	tr	tr			

Stains: R1^T^. *Duganella* sp. R1^T^; R64^T^. *Duganella* sp. R64^T^; HSC-15S17^T^. *Duganella violaceipulchra* HSC-15S17^T^; R57^T^. *Duganella* sp. R57^T^; KACC 21471^T^. *Duganella vulcania* KACC 21471^T^. All data were obtained from this study

^*^Summed Feature 3 comprised C16:1 ω7c and/or C16:1 ω6c; *Summed Feature 8 comprised C18:1 ω7c and/or C18:1 ω6c; *Summed Feature 9 comprised C17:1 iso ω9c and/or C16:0 10-methyl I

tr, Less than 1% of the total fatty acids

## Conclusion

The strains R1^T^, R57^T^, and R64^T^, isolated from barley roots, were identified as novel species within the genus *Duganella* based on their genomic, phylogenetic, phenotypic, and chemotaxonomic characterization. Accordingly, the names *Duganella hordei* sp. nov., *Duganella caerulea* sp. nov., and *Duganella rhizosphaerae* sp. nov. are proposed, respectively.

### Description of *Duganella hordei* sp. nov.

#### *Duganella hordei* (hor’de.i. L. gen. neut. n. *hordei* of/from barley, referring to the plant from which the type strain was isolated)

Cells are Gram-stain-negative, motile, and rod-shaped (approximately 0.5 µm × 1.3 µm in size). Colonies are white, smooth, flat, and circular on R2A agar. Growth occurs at 4–28 °C (optimum, 28 °C), pH 6.0–8.0 (optimum, pH 7.0), and 0–0.5% (w/v) NaCl (optimum, 0%). Other phenotypic and chemotaxonomic characteristics are provided in the text and in Supplementary Tables S9, S10, and S11. The G+C content of genomic DNA is 64.2%

The type strain, R1^T^ (= NBRC 115982^T^ = DSM 115069^T^), was isolated from barley roots in Okayama, Japan. The GenBank/EMBL/DDBJ accession numbers for the 16S rRNA gene sequence and draft genome are LC807588 and BPWI00000000, respectively.

### Description of *Duganella caerulea* sp. nov.

#### *Duganella caerulea* (cae.ru’le.a. L. fem. adj. *caerulea*, dark blue colored, referring to the colony color of the strain)

Cells are Gram-stain-negative, motile, and rod-shaped (approximately 0.8 µm × 1.7 µm in size). Colonies are purple, smooth, flat, and circular on R2A agar. Growth occurs at 4 to 28 °C (optimum, 28 °C), pH 6.0 to 8.0 (optimum, pH 7.0), and 0 to 0.5% (w/v) NaCl (optimum, 0%). Other phenotypic and chemotaxonomic characteristics are provided in the text and in Supplementary Tables S9, S10, and S11. The G+C content of genomic DNA is 64.7%

The type strain, R57^T^ (= NBRC 115983^T^ = DSM 115070^T^), was isolated from barley roots in Okayama, Japan. The GenBank/EMBL/DDBJ accession numbers for the 16S rRNA gene sequence and draft genome are LC807589 and BPWJ00000000, respectively.

### Description of *Duganella rhizosphaerae* sp. nov.

#### *Duganella rhizosphaerae* (rhi.zo.sphae’rae. N.L. gen. fem. n. *rhizosphaerae* of the rhizosphere, referring to the site from which the type strain was isolated)

Cells are Gram-stain-negative, motile, and rod-shaped (approximately 0.7 µm × 1.3 µm in size). Colonies are variably purple, smooth, flat, and circular on R2A agar. Growth occurs at 4–28 °C (optimum, 28 °C), pH 6.0 to 8.0 (optimum, pH 7.0), and 0 to 0.5% (w/v) NaCl (optimum, 0%). Other phenotypic and chemotaxonomic characteristics are provided in the text and in Supplementary Tables S9, S10, and S11. The G+C content of genomic DNA is 64.3%.

The type strain, R64^T^ (= NBRC 115984^T^ = DSM 115071^T^) was isolated from barley roots in Okayama, Japan. The GenBank/EMBL/DDBJ accession numbers for the 16S rRNA gene sequence and draft genome are LC807590 and BPWK00000000, respectively.

## Supplementary Information

Below is the link to the electronic supplementary material.

Supplementary file1 (PDF 1058 KB)

Supplementary file2 (XLSX 174 KB)

## Data Availability

The GenBank/EMBL/DDBJ accession numbers for the 16S rRNA gene sequences of strains R1T, R57T, and R64T are LC807588, LC807589, and LC807590, respectively. Those for the other isolates range from LC807591 to LC807690. The GenBank/EMBL/DDBJ accession numbers for the draft genomes of strains R1T, R57T, and R64T are BPWI00000000, BPWJ00000000, and BPWK00000000, respectively.

## Abbreviations

ICNP: International code of nomenclature of prokaryotes
LPSN: List of prokaryotic names with standing in nomenclature
R2A: Reasoner’s 2A
dDDH: Digital DNA-DNA hybridization
TYGS: Type (strain) genome server
ANIb: Average nucleotide identity by blast

## Declarations

### Human and animal participants

This article does not contain any studies with human or animal subjects.
