# Supplementary material for: Duganella hordei sp. nov., Duganella caerulea sp. nov., and Duganella rhizosphaerae sp. nov., isolated from barley rhizosphere
Source: Antonie Van Leeuwenhoek. 2025 Sep 1;118(10):146. doi: 10.1007/s10482-025-02160-2 (PMC12401772; doi:10.1007/s10482-025-02160-2)
Supplement: Supplementary file 1 — Supplementary file1 (PDF 1058 KB) [file 10482_2025_2160_MOESM1_ESM.pdf]

Supplementary information

Antonie van Leeuwenhoek

Duganella hordei sp. nov., *Duganella caerulea* sp. nov., and *Duganella rhizosphaerae* sp. nov., isolated from barley rhizosphere

Katsumoto Kishiro[1], Nurettin Sahin[2], Daisuke Saisho[1], Naoki Yamaji[1], Jun Yamashita[1], Yuki Monden[3], Tomoyuki Nakagawa[4], Keiichi Mochida[5,6], Akio Tani[1]

1 Institute of Plant Science and Resources, Okayama University, Kurashiki, Okayama, Japan
2 Egitim Facultesi, Mugla Sitki Kocman University, Mugla, Turkiye
3 Graduate School of Environmental, Life, Natural Science and Technology, Okayama University, Okayama, Japan
4 Faculty of Applied Biological Sciences, Gifu University, Gifu, Japan
5 RIKEN Center for Sustainable Resource Science, Yokohama, Kanagawa, Japan
6 School of Information and Data Sciences, Nagasaki University, Nagasaki, Japan

Corresponding author: Dr. Akio Tani
e-mail: atani@okayama-u.ac.jp

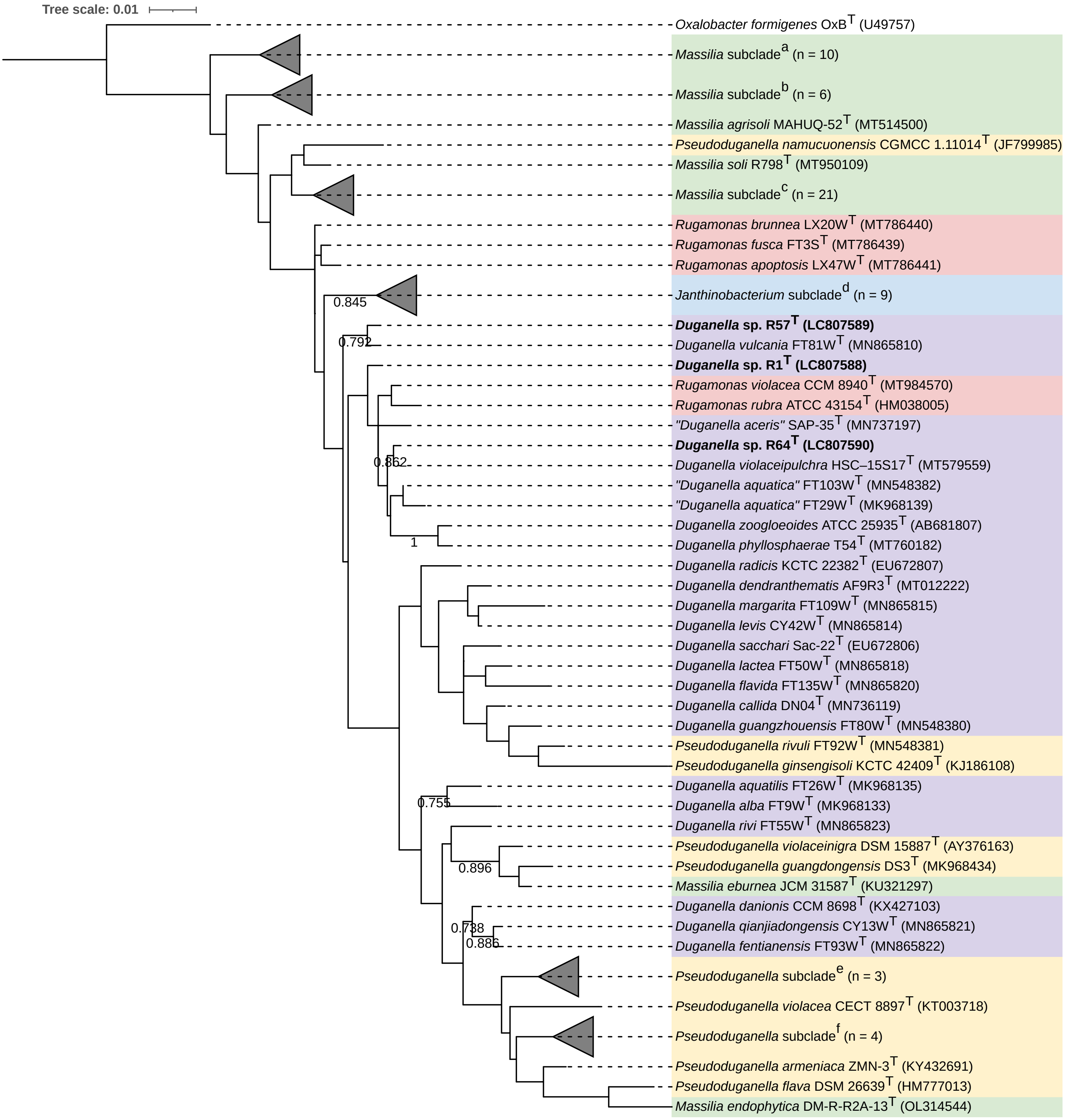

Fig. S1 Maximum-likelihood phylogenetic tree based on 16S rRNA gene sequences of R1^{T}, R57^{T}, R64^{T}, and their relative strains. *Oxalobacter formigenes* OxB^{T} is used as the outgroup.

Bootstrap values (0.7-1) of 1000 replicates are shown. Bar, 0.01 substitutions per nucleotide position. *Massilia* subclade[a] comprises *M. forsythiae* GN2-R2^{T} (MT012223), *M. rhizosphaerae* NEAU GH312^{T} (MW198488), *M. norwichensis* NS9^{T} (HG798294), *M. putida* 6NM-7^{T} (JQ608336), *M. pinisoli* T33^{T} (KU296190), *M. niastensis* 5516S-1^{T} (EU808005), *M. horti* ONC3^{T} (MT378212), *M. terrae* J11^{T} (KU870757), *M. solisilvae* J18^{T} (KU870758), and *M. agilis* J9^{T} (KU870756). *Massilia* subclade[b] comprises *M. consociata* CCM 7792^{T} (MT760179), *M. yuzhufengensis* Y1243-1^{T} (JQ409016), *M. phyllostachyos* G4R7^{T} (MZ573411), *M. agri* K-3-1^{T} (KX672812), *M. varians* CCUG 35299^{T} (AM774587), and *M. alkalitolerans* YIM 31775^{T} (AY679161).

Massilia subclade[c] comprises *M. jejuensis* 5317J-18^{T} (FJ969486), *M. brevitalea* byr23-80^{T} (EF546777), *M. niabensis* 5420S-26^{T} (EU808006), *M. suwonensis* 5414S-25^{T} (FJ969487), *M. haematophila* CCM 7480^{T} (MT758009), *M. aurea* CCM 7363^{T} (MT760134), *M. timonae* UR/MT95^{T} (U54470), *M. oculi* CCM 7900^{T} (MT760187), *M. puerhi* SJY3^{T} (MN014073), *M. arenae* GEM 5^{T} (KT369857), *M. polaris* RP-1-19^{T} (MN685328), *M. psychrophila* B1555-1^{T} (KM873051), *M. eurypsychrophila* B528-3^{T} (KJ361504), *M. glaciei* B448-2^{T} (KJ755877), *M. aquatica* CCM 8693^{T} (MN612031), *M. mucilaginosa* CCM 8733^{T} (MN612043), *M. atriviolacea* SOD^{T} (MH551481), *M. frigida* CCM 8695^{T} (MN612047), *M. antarctica* CCM 8941^{T} (OM243916), *M. violaceinigra* B 2^{T} (KF267246), and *M. rubra* CCM 8692^{T} (MN611986). *Janthinobacterium* subclade[d] comprises *J. psychrotolerans* S3-2^{T} (LOCQ01000055), *J. agaricidamnosum* W1r3^{T} (Y08845), *J. violaceinigrum* FT13W^{T} (MK968136), *J. fluminis* hw3^{T} (ON668161), *J. aquaticum* FT58W^{T} (MN548378), *J. kumbetense* GK^{T} (MZ434955), *J. rivuli* FT68W^{T} (MN548379), *J. tructae* SNU WT3^{T} (MN524134), and *J. lividum* DSMZ 1522^{T} (MN307292). *Pseudoduganella* subclade[e] comprises *P. buxea* CGMCC 1.15931^{T} (KX944690), *P. plicata* KCTC 12344^{T} (MT758059), and *P. lurida* CGMCC 1.10822^{T} (HQ839786). *Pseudoduganella* subclade[f] comprises *P. dura* DSM 17513^{T} (MT758057), *P. umbonata* DSM 26121^{T} (HM053474), *P. albidiflava* DSM 17472^{T} (MT758058), and *P. lutea* DSM 17473^{T} (AY966001).

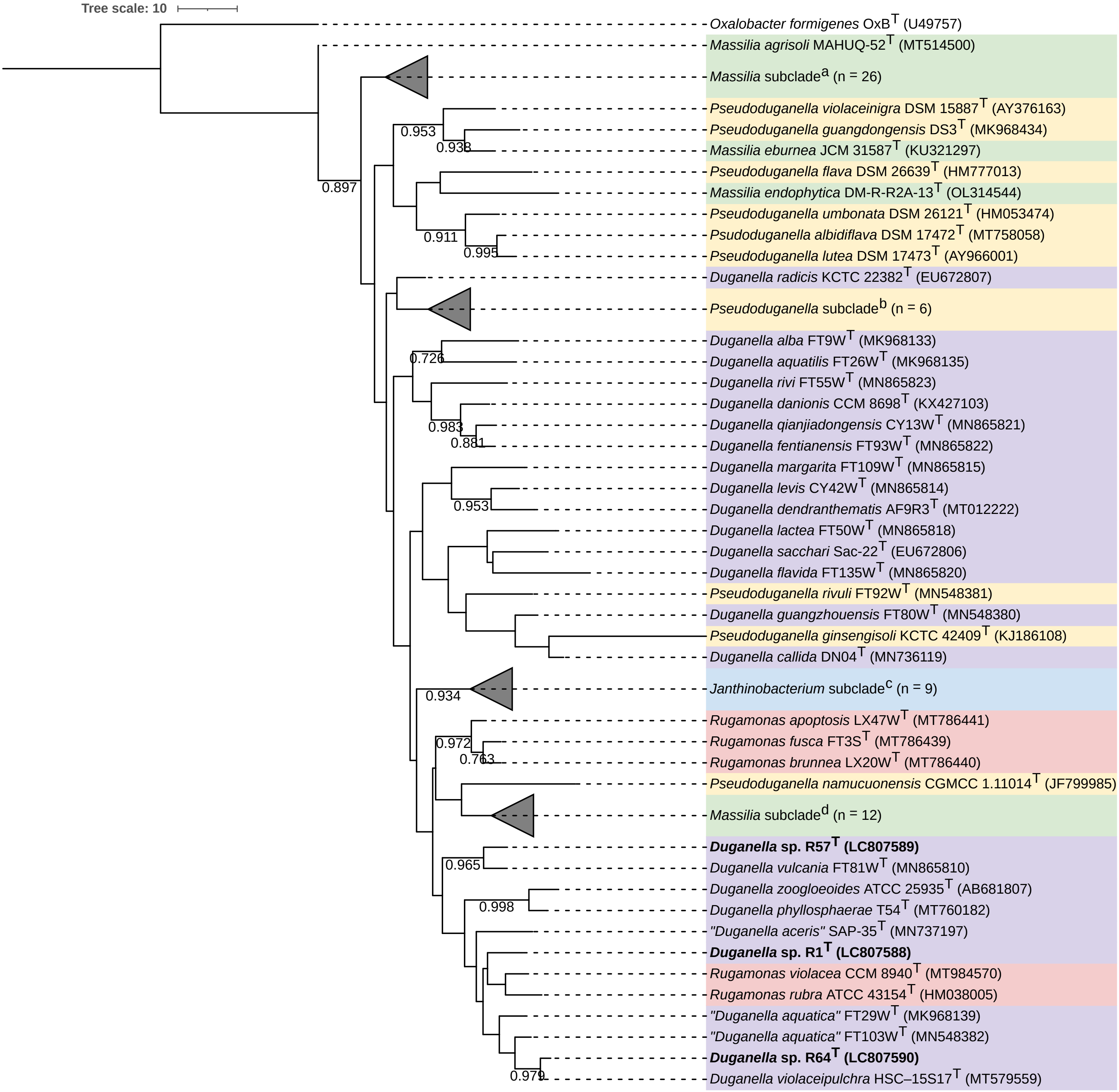

Fig. S2 Neighbor-joining phylogenetic tree based on 16S rRNA gene sequences of R1^{T}, R57^{T}, R64^{T}, and their relative strains. *Oxalobacter formigenes* OxB^{T} is used as the outgroup.

Bootstrap values (0.7-1) of 1000 replicates are shown. Bar, 10 substitutions per nucleotide position. *Massilia* subclade[a] comprises *M. horti* ONC3^{T} (MT378212), *M. terrae* J11^{T} (KU870757), *M. solisilvae* J18^{T} (KU870758), *M. agilis* J9^{T} (KU870756), *M. consociata* CCM 7792^{T} (MT760179), *M. agri* K-3-1^{T} (KX672812), *M. yuzhufengensis* Y1243-1^{T} (JQ409016), *M. phyllostachyos* G4R7^{T} (MZ573411), *M. pinisoli* T33^{T} (KU296190), *M. niastensis* 5516S-1^{T} (EU808005), *M. forsythiae* GN2-R2^{T} (MT012223), *M. putida* 6NM-7^{T} (JQ608336), *M. rhizosphaerae* NEAU GH312^{T} (MW198488), *M. norwichensis* NS9^{T} (HG798294), *M. suwonensis* 5414S-25^{T} (FJ969487), *M. haematophila* CCM 7480^{T} (MT758009), *M. varians* CCUG 35299^{T} (AM774587), *M. alkalitolerans* YIM 31775^{T} (AY679161), *M. niabensis* 5420S-26^{T} (EU808006), *M. aurea* CCM 7363^{T} (MT760134), *M. jejuensis* 5317J-18^{T} (FJ969486), *M. brevitalea* byr23-80^{T} (EF546777), *M. oculi* CCM 7900^{T} (MT760187), *M. timonae* UR/MT95^{T} (U54470), *M. puerhi* SJY3^{T} (MN014073), and *M. arenae* GEM 5^{T} (KT369857). *Pseudoduganella* subclade[b] comprises *P. violacea* CECT 8897^{T} (KT003718), *P. armeniaca* ZMN-3^{T} (KY432691), *P. lurida* CGMCC 1.10822^{T} (HQ839786), *P. dura* DSM 17513^{T} (MT758057), *P. plicata* KCTC 12344^{T} (MT758059), and *P. buxea* CGMCC 1.15931^{T} (KX944690). *Janthinobacterium* subclade[c] comprises *J. fluminis* hw3^{T} (ON668161), *J. psychrotolerans* S3-2^{T} (LOCQ01000055), *J. agaricidamnosum* W1r3^{T} (Y08845), *J. violaceinigrum* FT13W^{T} (MK968136), *J. aquaticum* FT58W^{T} (MN548378), *J. tructae* SNU WT3^{T} (MN524134), *J. kumbetense* GK^{T} (MZ434955), *J. rivuli* FT68W^{T} (MN548379), and *J. lividum* DSMZ 1522^{T} (MN307292). *Massilia* subclade[d] comprises *M. soli* R798^{T} (MT950109), *M. polaris* RP-1-19^{T} (MN685328), *M. psychrophila* B1555-1^{T} (KM873051), *M. eurypsychrophila* B528-3^{T} (KJ361504), *M. glaciei* B448-2^{T} (KJ755877), *M. mucilaginosa* CCM 8733^{T} (MN612043), *M. atriviolacea* SOD^{T} (MH551481), *M. aquatica* CCM 8693^{T} (MN612031), *M. antarctica* CCM 8941^{T} (OM243916), *M. frigida* CCM 8695^{T} (MN612047), *M. violaceinigra* B 2^{T} (KF267246), and *M. rubra* CCM 8692^{T} (MN611986).

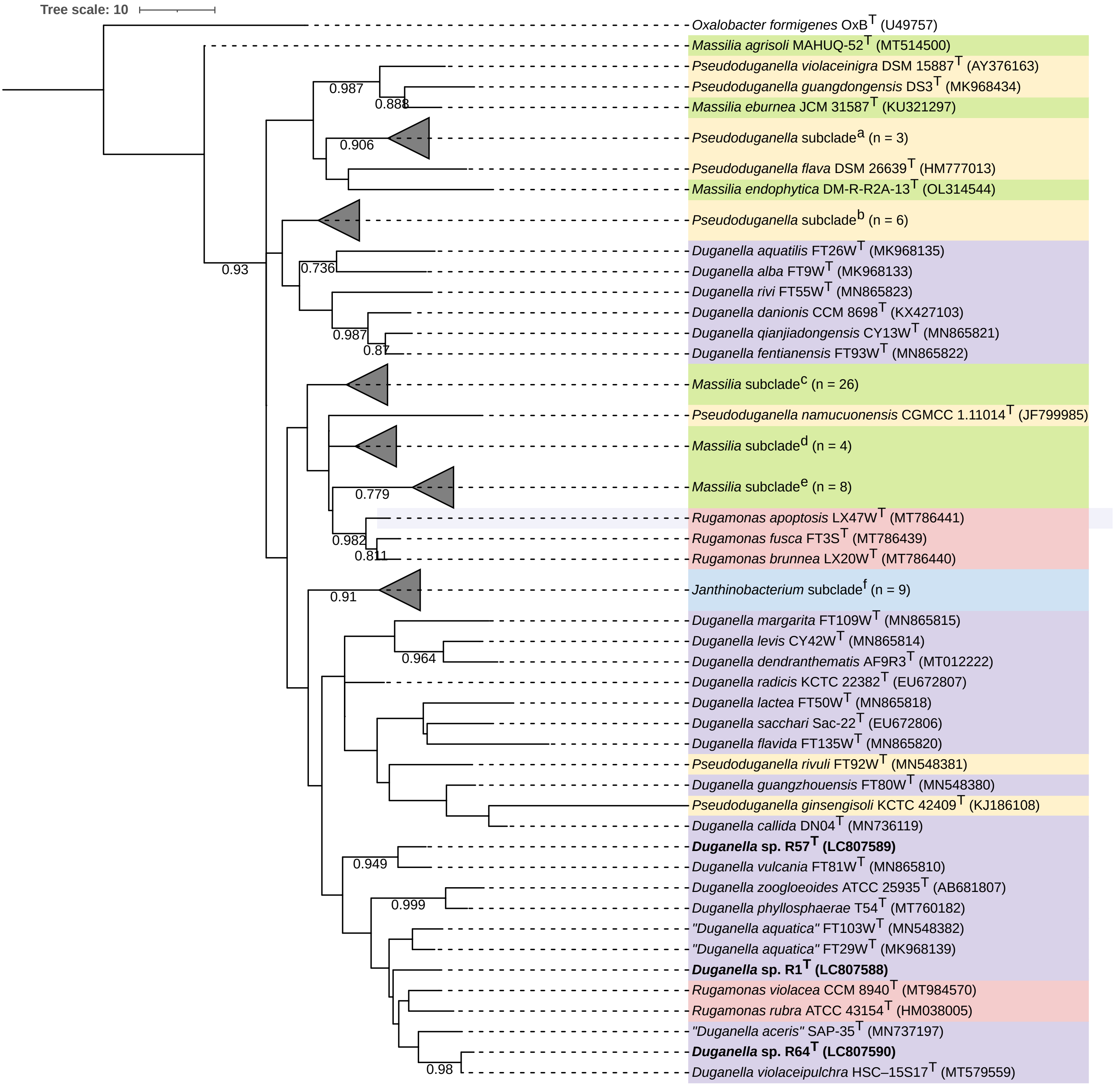

Fig. S3 Minimum-evolution phylogenetic tree based on 16S rRNA gene sequences of R1^{T}, R57^{T}, R64^{T}, and their relative strains. *Oxalobacter formigenes* OxB^{T} is used as the outgroup.

Bootstrap values (0.7-1) of 1000 replicates are shown. Bar, 10 substitutions per nucleotide position. *Pseudoduganella* subclade[a] comprises *P. umbonata* DSM 26121^{T} (HM053474), *P. albidiflava* DSM 17472^{T} (MT758058), and *P. lutea* DSM 17473^{T} (AY966001). *Pseudoduganella* subclade[b] comprises *P. violacea* CECT 8897^{T} (KT003718), *P. armeniaca* ZMN-3^{T} (KY432691), *P. lurida* CGMCC 1.10822^{T} (HQ839786), *P. dura* DSM 17513^{T} (MT758057), *P. plicata* KCTC 12344^{T} (MT758059), and *P. buxea* CGMCC 1.15931^{T} (KX944690). *Massilia* subclade[c] comprises *M. niabensis* 5420S-26^{T} (EU808006), *M. aurea* CCM 7363^{T} (MT760134), *M. jejuensis* 5317J-18^{T} (FJ969486), *M. brevitalea* byr23-80^{T} (EF546777), *M. oculi* CCM 7900^{T} (MT760187), *M. timonae* UR/MT95^{T} (U54470), *M. puerhi* SJY3^{T} (MN014073), *M. arenae* GEM 5^{T} (KT369857), *M. suwonensis* 5414S-25^{T} (FJ969487), *M. haematophila* CCM 7480^{T} (MT758009), *M. varians* CCUG 35299^{T} (AM774587), *M. alkalitolerans* YIM 31775^{T} (AY679161), *M. yuzhufengensis* Y1243-1^{T} (JQ409016), *M. phyllostachyos* G4R7^{T} (MZ573411), *M. consociata* CCM 7792^{T} (MT760179), *M. agri* K-3-1^{T} (KX672812), *M. horti* ONC3^{T} (MT378212), *M. terrae* J11^{T} (KU870757), *M. solisilvae* J18^{T} (KU870758), *M. agilis* J9^{T} (KU870756), *M. pinisoli* T33^{T} (KU296190), *M. niastensis* 5516S-1^{T} (EU808005), *M. forsythiae* GN2-R2^{T} (MT012223), *M. putida* 6NM-7^{T} (JQ608336), *M. rhizosphaerae* NEAU GH312^{T} (MW198488), and *M. norwichensis* NS9^{T} (HG798294). *Massilia* subclade[d] comprises *M. soli* R798^{T} (MT950109), *M. polaris* RP-1-19^{T} (MN685328), *M. psychrophila* B1555-1^{T} (KM873051), and *M. eurypsychrophila* B528-3^{T} (KJ361504). *Massilia* subclade[e] comprises *M. glaciei* B448-2^{T} (KJ755877), *M. mucilaginosa* CCM 8733^{T} (MN612043), *M. atriviolacea* SOD^{T} (MH551481), *M. aquatica* CCM 8693^{T} (MN612031), *M. antarctica* CCM 8941^{T} (OM243916), *M. frigida* CCM 8695^{T} (MN612047), *M. violaceinigra* B 2^{T} (KF267246), and *M. rubra* CCM 8692^{T} (MN611986). *Janthinobacterium* subclade[f] comprises *J. fluminis* hw3^{T} (ON668161), *J. agaricidamnosum* W1r3^{T} (Y08845), *J. violaceinigrum* FT13W^{T} (MK968136), *J. aquaticum* FT58W^{T} (MN548378), *J. psychrotolerans* S3-2^{T} (LOCQ01000055), *J. tructae* SNU WT3^{T} (MN524134), *J. kumbetense* GK^{T} (MZ434955), *J. rivuli* FT68W^{T} (MN548379), and *J. lividum* DSMZ 1522^{T} (MN307292).

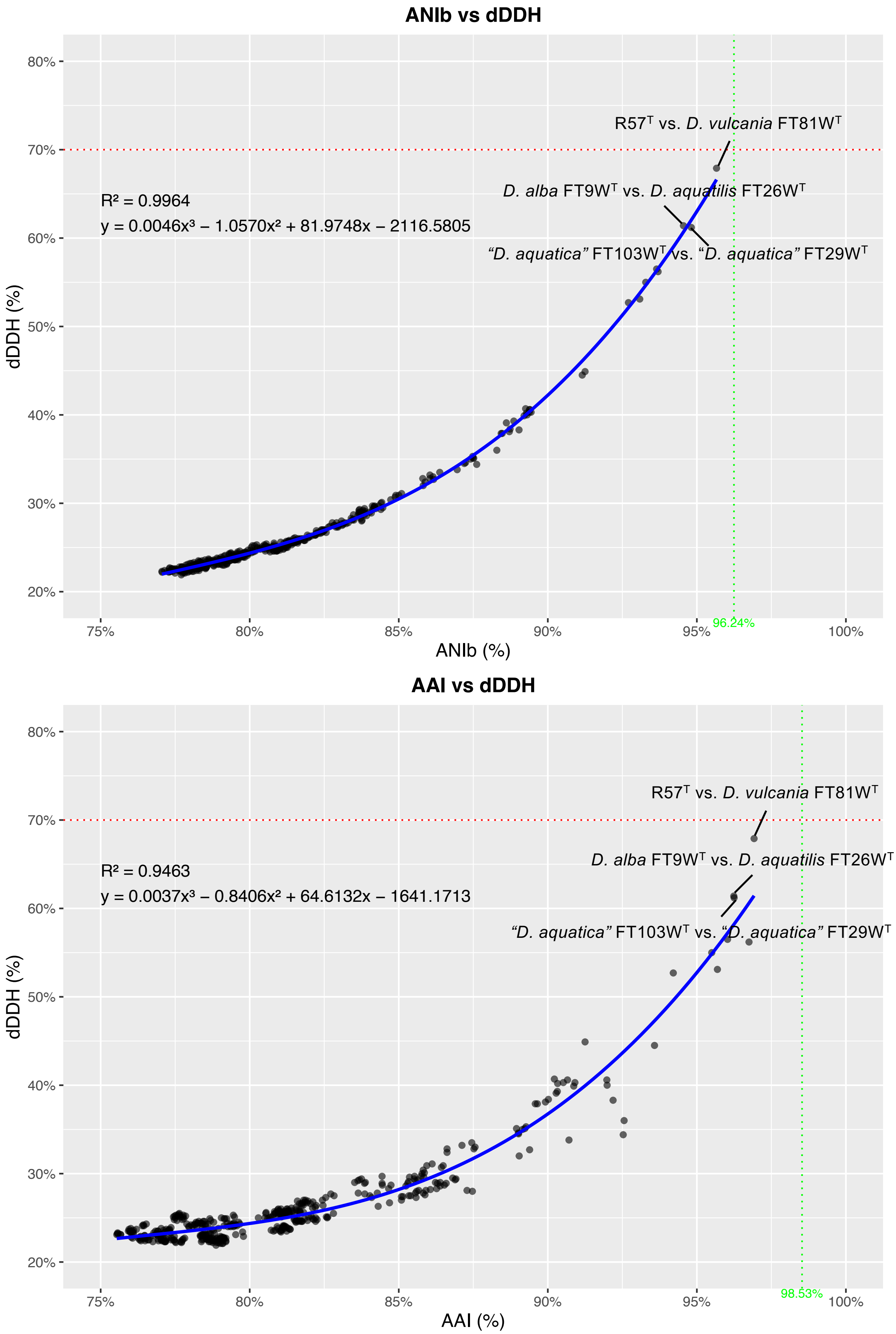

Figure S4 The correlations between ANIb, dDDH, from the 435 pairs of *Duganella* and *Rugamonas* species (including synonyms). The data (Table S5) are graphed. The bidirectional ANIb values are averaged. The correlation was analyzed using a quadratic regression, and the regression equations and R-squared values are shown within the graphs.

Duganella sp. R1^{T}

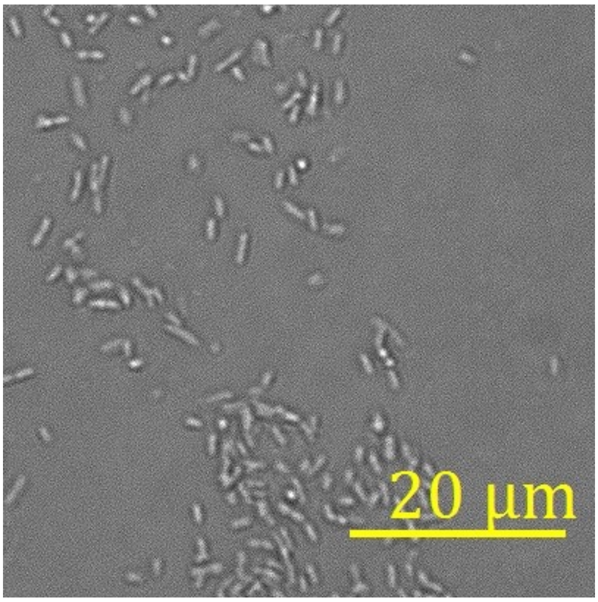

Duganella sp. R57^{T}

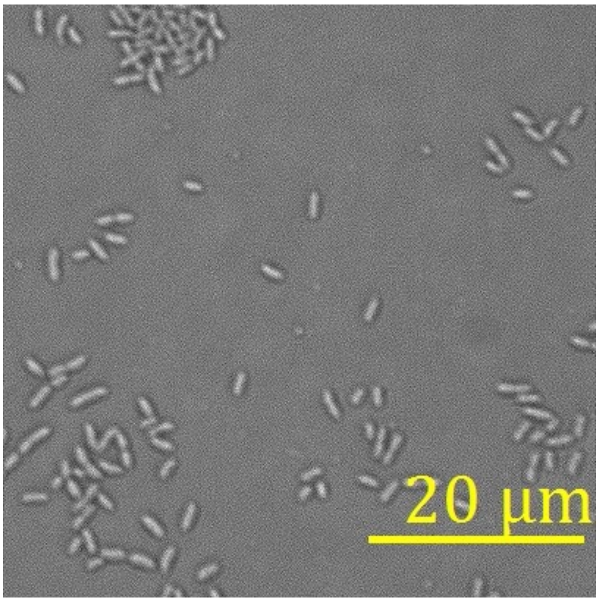

Duganella sp. R64^{T}

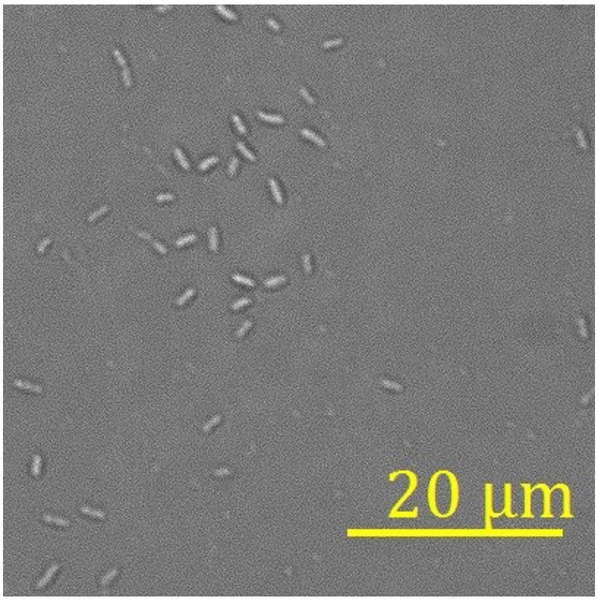

D. violaceipulchra HSC-15S17^{T}

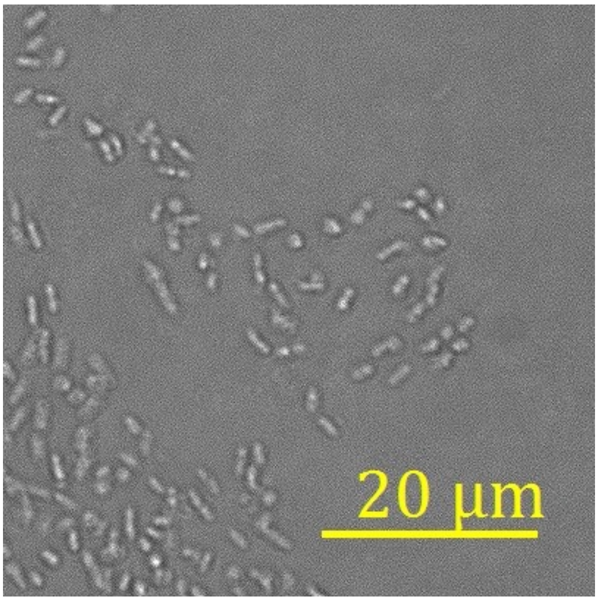

D. vulcania KACC 21471^{T}

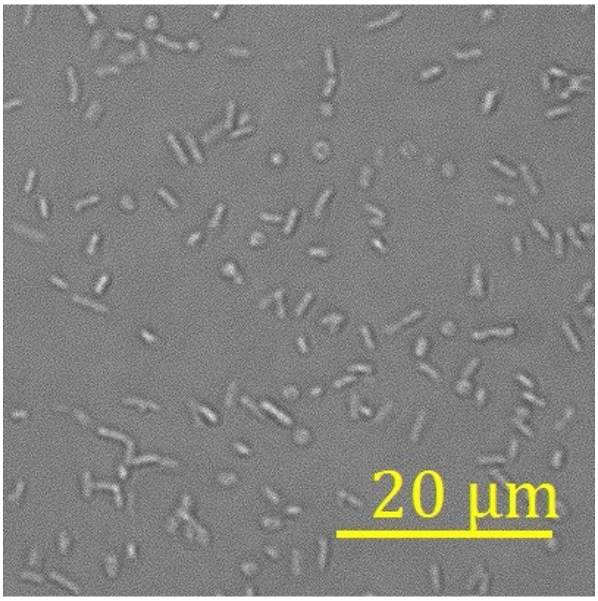

Figure S5 Microscopic images of strains R1^{T}, R57^{T}, R64^{T}, HSC-15S17^{T}, and KACC 21471^{T}
Cells were grown in R2A broth at 28°C for 2 days. Microscopic observations were performed using a fluorescence microscope (BZ-X700, Keyence, Japan) with a 111x immersion objective.

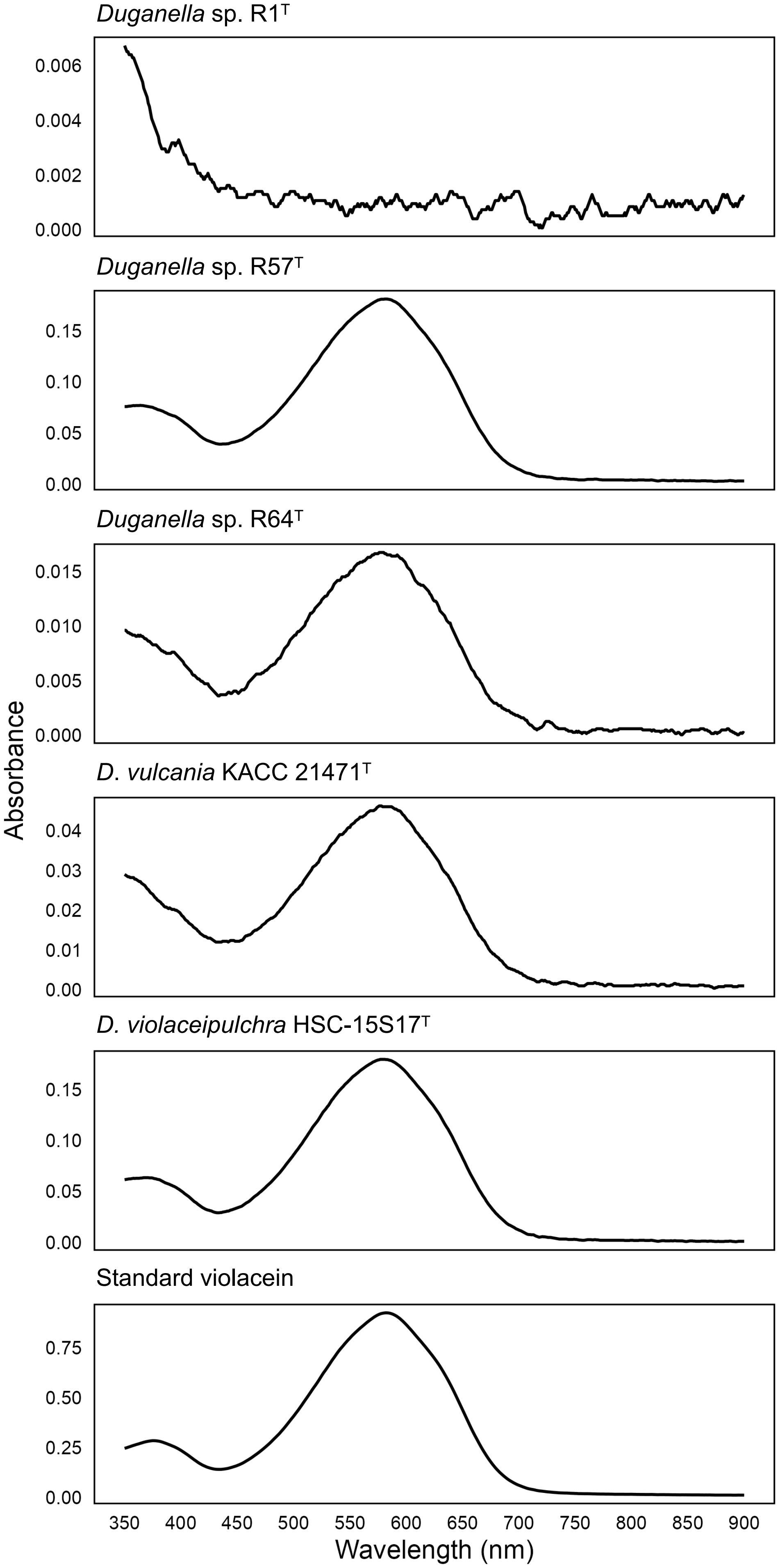

Figure S6 Absorption spectra of 1-butanol extracts of R1^T, R57^T, R64^T, KACC 21471^T, HSC-15S17^T, and standard violacein. Standard 0.1 mg/mL violacein (Cayman Chemical, USA) was dissolved in 1-butanol. The extracts were obtained by extracting strains with 1-butanol. Spectra were recorded using a microplate reader (DS Pharma Biomedical). All spectra, except that of *Duganella* sp. R1^T, exhibited absorption peaks at approximately 583 nm. *Duganella* sp. R1^T showed no detectable peak.
